# Unusual Presentation of Unilateral Isolated Probable Lyme Optic Neuritis

**DOI:** 10.1155/2016/7471842

**Published:** 2016-02-03

**Authors:** Ahmet Z. Burakgazi, Carl S. Henderson

**Affiliations:** ^1^Internal Medicine, Neurology Department, Virginia Tech Carilion School of Medicine and Research Institute, Roanoke, VA 24016, USA; ^2^Internal Medicine, Rheumatology Department, Virginia Tech Carilion School of Medicine and Research Institute, Roanoke, VA 24016, USA

## Abstract

Optic neuritis (ON) is one of the most common manifestations of central nervous system involvement caused by various etiologies. Lyme ON is an exceedingly rare ocular manifestation of Lyme disease (LD) and only a few cases have been published in the literature. Lyme ON is very rare but should be included in the differential diagnosis in unexplained cases, particularly in Lyme endemic areas. Careful and detailed examination and investigation are warranted to make the diagnosis. We report this case to increase awareness of clinicians to include Lyme disease in differential diagnosis of ON for unexplained cases of ON. Herein we present a unique case with a unilateral ON caused by LD along with pre- and posttreatment findings and literature review.

## 1. Introduction

Optic neuritis (ON) is an inflammation of the optic nerve and one of the most common manifestations of central nervous system involvement caused by various etiologies [1–5]. Well-descried causes of optic neuritis include demyelinating diseases [4, 5], autoimmune diseases [2–4], inflammatory diseases, infections, and vaccinations. The cardinal signs of ON are decreased visual acuity, a central visual field defect, dyschromatopsia, and a relative afferent pupil defect [2–5].

Lyme disease (LD), the leading arthropod-borne infection in the northern hemisphere, can cause a wide spectrum of neurological conditions involving central and peripheral nervous systems [6–8]. LD-related ocular complications are uncommon, but various types of manifestations have been described including conjunctivitis, keratitis, and extra-ocular muscle palsies [8, 9]. Lyme ON is an exceedingly rare ocular manifestation of LD and only a few cases have been published in the literature.

Herein, we report a unique case with a unilateral ON caused by LD along with pre- and posttreatment findings and literature review.

## 2. Case Presentation

A 59-year-old female with a recent past medical history of mesentery thrombosis on stable anticoagulation and minimal other past medical history was referred to our clinic from ophthalmology clinic for unexplained decreased right eye visual acuity and visual field defect and right optic disc swelling. She presented with approximately 3-4 weeks history of right eye vision changes and seen by rheumatologist after she was noted to have a positive ANA. She had right blurry vision that worsened with focusing on near objects. One to two weeks after having vision symptoms, she noticed papular erythematous blanching rash across the shoulders extending to midback, part of chest, left shoulder, and right cheek. She also described tiredness and generalized joints pain. She denied any other neurological or systemic symptoms. She did not recall any tick bite. She did not have similar episodes in the past. She did not have any other medical diseases. She did not take any other medications before her symptoms and any medications on regular basis. Her family history was unremarkable.

Her physical examination showed optic nerve swelling on the right eye and mild afferent pupil defect on the right eye. Visual acuity with Snellen Eye Test Charts was 20/30 on the right eye and 20/20 with the left eye. The remainder of her neurological examination, including cranial nerves, motor exam, sensory exam, reflexes, coordination, and gait, was within normal limits. No meningeal signs such as neck stiffness were detected.

**Figure 1:**
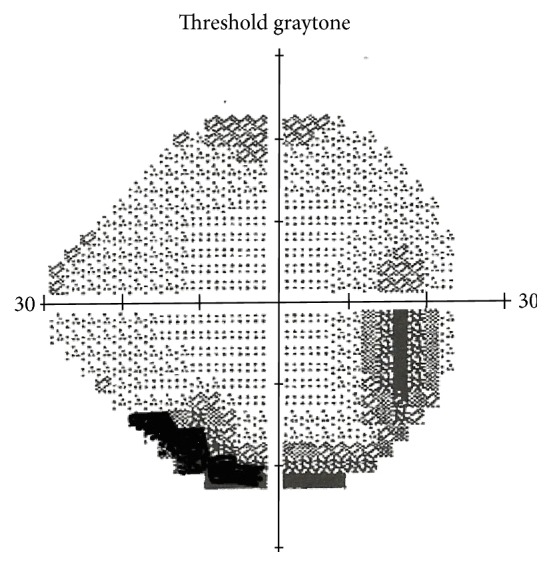
An automated perimetry visual field test before the treatment demonstrated arcuate scotoma, inferior partial arcuate defect, and superior vertical step defect that indicated optic nerve fiber bundle involvement.

She underwent detailed investigation including biochemical tests, magnetic resonance imaging (MRI) of brain, magnetic resonance venography (MRV) of head, and lumbar puncture. The cerebrospinal fluid examination showed normal cell count (white blood cell count: 4; and red blood cell count: 3), mildly elevated protein 55 mg/dL (normal range 15–45 mg/dL), elevated Lyme disease IgG 1 : 32 (reference ranges IgG < 1 : 4), and positive oligoclonal bands (>5 well defined gamma restriction bands). She underwent extensive blood tests which showed positive Lyme Western Blot with bands (positive seven of 10 IgG bands; positive three of three IgM bands); and normal results for other tests including C-reactive protein, sedimentation rate, antinuclear antibodies, 14-3-3 eta protein, C3 complement, C4 complement, total complement, CCP Antibody, rheumatoid factor, quantiferon-TB, HIV 1,2 AB, Hepatitis C antibody, Hepatitis B surface antigen, Hepatitis B surface antibody, cardiolipin antibodies, beta-s glycoprotein 1 antibodies IgA/IgM, and lupus anticoagulant panel. An automated perimetry visual field test before the treatment demonstrated arcuate scotoma, inferior partial arcuate defect, and superior vertical step defect that indicated optic nerve fiber bundle involvement (Figure 1). She was consulted to dermatology for the rash. The biopsy suggested granuloma annulare, in an interstitial pattern as seen with infections such as LD.

**Figure 2:**
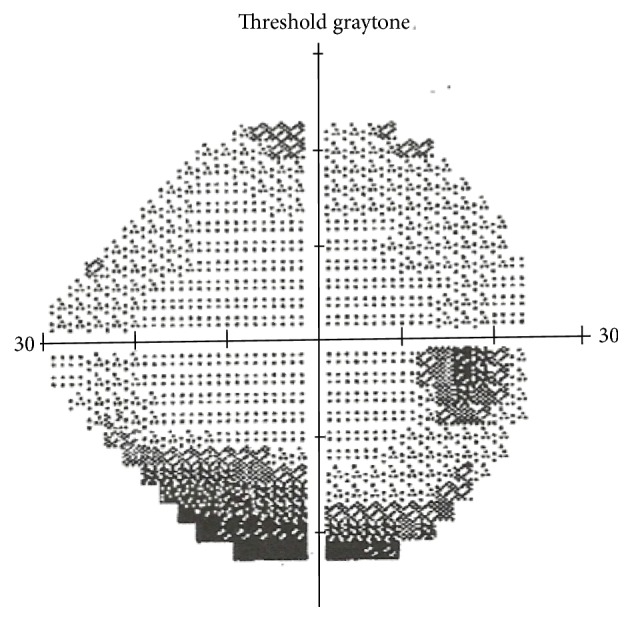
The follow-up perimetry visual field test after six-week doxycycline treatment demonstrated improvements of arcuate scotoma, superior vertical step, and inferior partial arcuate defects.

She was treated with doxycycline 100 mg twice a day for 6 weeks. She noticed some degree of improvement in her vision at the middle of the treatment, and she felt significant recovery at the end of the treatment. With the treatment, she felt more energy, less tiredness, and less joint pain. The follow-up ophthalmological examination showed decreased optic disc edema and improved visual acuity. Visual acuity with Snellen Eye Test Charts was 20/20 on the right eye and 20/20 with the left eye. The follow-up perimetry visual field test after six-week doxycycline treatment demonstrated improvements of arcuate scotoma, superior vertical step, and inferior partial arcuate defects (Figure 2).

## 3. Discussion

In this report, we present a case with isolated unilateral Lyme ON that is an extremely rare complication of LD. In our case, anti-Borrelia antibodies were present in serum and CSF and favorable outcome was achieved with six-week treatment of doxycycline. Further investigations were performed to rule out other possible etiologies of optic neuritis before making the final diagnosis. The patient also had some vague symptoms including arthralgia and skin rash that improved with doxycycline treatment.

Lyme ON is a rare complication of LD [10]. A few cases of Lyme ON have been reported in the literature. Blanc et al. reported two cases of isolated Lyme ON. Both patients had positive serum and cerebrospinal fluid serology, a positive intrathecal anti-Borrelia antibody index, and a good outcome on ceftriaxone [11]. Scott et al. reported a 10-year-old girl presenting with chiasmal ON whose history, clinical course, and increased serum Lyme immunoglobulin G titer were consistent with Lyme disease that improved with antibiotic treatment [12]. The last case of Lyme ON was reported in 2010. Although few isolated Lyme ON cases were reported in the literature, Kubová et al.'s cohort study including 81 patients with neuroborreliosis showed 42% had blurred vision or diplopia and 27% had a delayed visual evoked potential that means Lyme related optic nerve involvement may be more common than previously assumed [13]. We encountered this unusual presentation of ON, and all other etiological reasons were ruled out before making the final diagnosis of Lyme ON.

Ceftriaxone, cefotaxime, penicillin G, and doxycycline are highly effective in most neurological Lyme cases [6, 7, 14–17]. Intravenous ceftriaxone, high-dose penicillin, and cefotaxime have been used in patients with severe disease, particularly nervous system infection, and in patients who have not responded to oral regimens. European studies [6, 14, 15, 18–22] have shown that oral doxycycline, which achieves spirochetocidal concentration in the central nervous system (CNS), is highly effective in Lyme meningitis, cranial neuritis, and radiculoneuritis. In our case, the patient had a favorable improvement with oral doxycycline treatment; most of the reported Lyme ON cases were treated with ceftriaxone [11, 12, 16, 17, 23, 24].

The other unique part of our case was a demonstration of changes in visual field test before and after doxycycline treatment. We showed some improvements in visual field test with six-week doxycycline treatment. Thus, the visual field test can be used to assess the effectiveness of antibiotic treatment in Lyme ON.

A causal link between Lyme seropositivity and isolated ON is controversial. A few conflictive studies are available to discuss the relationship between LD and ON [24, 25]. Jacobson et al. [25] investigated 20 consecutive patients with newly diagnosed isolated optic neuritis who resided in a region endemic for LD; four patients had positive serum serology for LD. Three of these patients underwent CSF analysis and two had CSF lymphocytic pleocytosis that remained unexplained after extensive evaluations for causes other than LD. Two patients with CSF pleocytosis were treated with intravenous ceftriaxone and the other two received oral antibiotic treatment. Four of the patients had favorable recovery with the treatments [25]. In another study, Sibony et al. [24] retrospectively reviewed Lyme serology in 440 patients with ON examined between 1993 and 2003. They demonstrated that Lyme enzyme-linked immunosorbent assay (ELISA) was positive in 28 (6.4%) patients with optic neuritis, three of whom had syphilis with cross-reactive antibodies. Of the remaining 25 ELISA-positive patients, only one had Lyme disease-related optic neuritis and the other 24 cases had reactive Lyme serologies related to a history of LD years earlier, asymptomatic exposure, false-positive results, or nonspecific humoral expansion. Western blots confirmatory tests were negative in those 24 cases with reactive Lyme serologies. It was concluded that there is insufficient evidence for a causal link between LD and ON or neuroretinitis [24]. In our case, comprehensive investigation was performed to rule out other more common causes of optic neuritis [2–5, 11, 23, 26]. After ruling out the other more common reason, we included Lyme disease in the differential diagnosis.

## 4. Conclusion

In conclusion, ON is one of the most common manifestations of nervous system involvement caused by several autoimmune, inflammatory, and infection diseases. Lyme ON is rare but should be included in the differential diagnosis in unexplained cases, particularly in Lyme endemic areas. Careful and detailed examination and investigation are warranted to make the diagnosis. We report this case to increase awareness of clinicians to include Lyme disease in differential diagnosis of ON for unexplained cases of ON.
